# Safety and Efficacy of Low-Dose Tirofiban Combined With Intravenous Thrombolysis and Mechanical Thrombectomy in Acute Ischemic Stroke: A Matched-Control Analysis From a Nationwide Registry

**DOI:** 10.3389/fneur.2021.666919

**Published:** 2021-06-10

**Authors:** Gaoting Ma, Shuo Li, Baixue Jia, Dapeng Mo, Ning Ma, Feng Gao, Xiaochuan Huo, Gang Luo, Anxin Wang, Yuesong Pan, Ligang Song, Xuan Sun, Xuelei Zhang, Liqiang Gui, Cunfeng Song, Ya Peng, Jin Wu, Shijun Zhao, Junfeng Zhao, Zhiming Zhou, Zhongrong Miao

**Affiliations:** ^1^Interventional Neuroradiology Center, Beijing Tiantan Hospital, Capital Medical University, Beijing, China; ^2^Department of Neurology, Beijing Tiantan Hospital, Capital Medical University, Beijing, China; ^3^Department of Interventional Neuroradiology, Langfang Changzheng Hospital, Langfang, China; ^4^Department of Interventional Neuroradiology, Liao Cheng the Third People's Hospital, Liaocheng, China; ^5^Department of Neurosurgery, The First People's Hospital of Changzhou, The Third Affiliated Hospital of Soochow University, Changzhou, China; ^6^Department of Neurology, The Second Affiliated Hospital of Nanjing Medical University, Nanjing, China; ^7^Department of Interventional Radiology, Fengrun District People's Hospital of Tangshan City, Tangshan, China; ^8^Department of Neurology, SiPing Central People's Hospital, Siping, China; ^9^Department of Neurology, Yijishan Hospital of Wannan Medical College, Wuhu, China

**Keywords:** tirofiban, mechanical thrombectomy, intravenous thrombolysis, large vessel occlusion, propensity score matching

## Abstract

**Purpose:** Tirofiban administration to acute ischemic stroke patients undergoing mechanical thrombectomy with preceding intravenous thrombolysis remains controversial. The aim of the current study was to evaluate the safety and efficacy of low-dose tirofiban during mechanical thrombectomy in patients with preceding intravenous thrombolysis.

**Methods:** Patients with acute ischemic stroke undergoing mechanical thrombectomy and preceding intravenous thrombolysis were derived from “ANGEL-ACT,” a multicenter, prospective registry study. The patients were dichotomized into tirofiban and non-tirofiban groups based on whether tirofiban was administered. Propensity score matching was used to minimize case bias. The primary safety endpoint was symptomatic intracerebral hemorrhage (sICH), defined as an intracerebral hemorrhage (ICH) associated with clinical deterioration as determined by the Heidelberg Bleeding Classification. All ICHs and hemorrhage types were recorded. Clinical outcomes included successful recanalization, dramatic clinical improvement, functional independence, and mortality at the 3-month follow-up timepoint. Successful recanalization was defined as a modified Thrombolysis in Cerebral Ischemia score of 2b or 3. Dramatic clinical improvement at 24 h was defined as a reduction in NIH stroke score of ≥10 points compared with admission, or a score ≤1. Functional independence was defined as a Modified Rankin Scale (mRS) score of 0–2 at 3-months.

**Results:** The study included 201 patients, 81 in the tirofiban group and 120 in the non-tirofiban group, and each group included 68 patients after propensity score matching. Of the 201 patients, 52 (25.9%) suffered ICH, 15 (7.5%) suffered sICH, and 18 (9.0%) died within 3-months. The median mRS was 3 (0–4), 99 (49.3%) achieved functional independence. There were no statistically significant differences in safety outcomes, efficacy outcomes on successful recanalization, dramatic clinical improvement, or 3-month mRS between the tirofiban and non-tirofiban groups (all *p* > 0.05). Similar results were obtained after propensity score matching.

**Conclusion:** In acute ischemic stroke patients who underwent mechanical thrombectomy and preceding intravenous thrombolysis, low-dose tirofiban was not associated with increased risk of sICH or ICH. Further randomized clinical trials are needed to confirm the effects of tirofiban in patients undergoing bridging therapy.

## Introduction

Endovascular treatment has proved to be effective for improving functional outcomes and reducing mortality in patients with large-artery occlusive stroke (1–7). However, during the operative procedure, platelet aggregation caused by severe atherosclerotic stenosis or endothelial damage can lead to thrombotic events and early re-occlusion (8, 9). The highly selective glycoprotein IIb/IIIa receptor antagonist tirofiban can efficiently block the final pathway of platelet aggregation and subsequent thrombus formation (10).

A number of studies have reported the effects of tirofiban during mechanical thrombectomy (MT), but outcomes are controversial (11–14). One of the main concerns is whether tirofiban will lead to increased risks of bleeding in patients who have received intravenous thrombolysis (IVT) before MT. Because of this, the use of antiplatelet agents is not recommended within 24 h after IVT in the American Heart Association/American Stroke Association (AHA/ASA) guidelines (15). Few prospective studies have focused on tirofiban administration during MT in patients with preceding IVT, which is also known as bridging therapy. The aim of the current prospective multicenter study was to evaluate the safety of tirofiban during MT with respect to symptomatic intracerebral hemorrhage (sICH) and intracerebral hemorrhage (ICH), as well as its efficacy during artery recanalization, and functional outcomes in patients who underwent bridging IVT.

## Materials and Methods

### Patient Enrolment

All patients were enrolled from the registry of “ANGEL-ACT,” which was a nationwide, multicenter, prospective registry study conducted in China from November 2017 to March 2019. Details of the design of the ANGEL-ACT have been reported previously (16). The protocol of the ANGEL-ACT was approved by the Ethics Committee of Beijing Tiantan Hospital and all other participating centers. Written informed consent was obtained from all patients or their representatives. The current study included the following data: (1) Anterior circulation large vessel occlusion (ICA/M1); (2) onset to groin time ≤6 h; (3) the Alberta Stroke Program Early CT Score (ASPECTS) ≥6; and (4) underwent thrombolytic therapy. The main exclusion criterion was incomplete clinical data.

### Endovascular Interventions and Grouping

In all MTs a stent retriever or aspiration device was the first recanalization option, in accordance with protocol. In cases in which the first recanalization failed, additional thrombectomy attempts and alternative rescue therapies were used at the discretion of the operator, including intra-arterial or intravenous tirofiban administration, intra-arterial thrombolysis, balloon angioplasty, and emergent stenting. The patients were divided into a tirofiban group and a non-tirofiban group based on tirofiban administration during MT.

### Tirofiban Administration During Mechanical Thrombectomy

All eligible patients underwent endovascular treatment immediately after the assessment of indications. In general, tirofiban was given under the following conditions: (1) Emergency stenting for severe residual stenosis or instant re-occlusion; (2) balloon angioplasty for severe residual stenosis or instant re-occlusion; (3) successful mechanical recanalization with three or more passes with a stent retriever for presumed endothelial damage or instant re-occlusion; and (4) severe *in situ* atherosclerosis with a high risk of early re-occlusion. Unless an ICH was suspected, a low-dose intra-arterial bolus (0.25–1.00 mg) followed by a continuous intravenous infusion (0.1 μg/kg/min) was administrated for 24 h as a standard procedure. At 4 h prior to the end of the infusion, dual antiplatelet agents (aspirin 100 mg and clopidogrel 75 mg) were administered as bridging therapy if ICH was excluded within 24 h via follow-up computed tomography or magnetic resonance imaging.

### Safety and Efficacy Outcomes

The main safety endpoints were sICH, ICH, and mortality within 3-months. sICH was defined as an ICH associated with clinical deterioration according to the Heidelberg Bleeding Classification (17). Hemorrhage types were also recorded. Hemorrhagic outcomes were assessed by a core laboratory, blinded to the clinical data and outcomes. Efficacy outcomes included successful recanalization, dramatic clinical improvement, and functional independence. Successful recanalization was defined as a modified Thrombolysis in Cerebral Ischemia (mTICI) score of 2b or 3 (18). Dramatic clinical improvement was defined at 24 h as a reduction in NIH Stroke Scale (NIHSS) score of ≥ 10 points compared with admission, or a score of ≤1 (19). Functional independence was defined as a modified Rankin Scale (mRS) score of 0–2 at 3-months.

### Statistical Analysis

Baseline patient demographic information in the tirofiban and non-tirofiban groups were compared, as were all endpoints. A logistic regression model was used to investigate associations between tirofiban administration and safety and efficacy endpoints. To reduce data bias and confounding variables, propensity score matching (PSM) analysis was performed by matching patients in the two groups at a 1:1 ratio. Age, sex, baseline Modified Rankin Scale score, baseline NIHSS score, ASPECTS score, onset-to-puncture time, and pathogenesis of stroke were used to generate a propensity score for each subject. After PSM the two groups were again compared via the aforementioned statistical methods.

For continuous data, means ± standard deviation or medians and interquartile ranges were used to summarize data, and two-sided *t*-tests for independent samples or Mann-Whitney U tests were used to assess the significance of differences between groups. Frequencies and percentages were used to summarize binary data, and between-group comparisons were performed via the χ2 test or Fisher's exact tests as appropriate. All analyses were performed using SAS version 9.4 software (SAS Institute, Cary, NC, USA), and *p* < 0.05 was considered statistically significant.

## Results

### Baseline Characteristics

**Figure 1:**
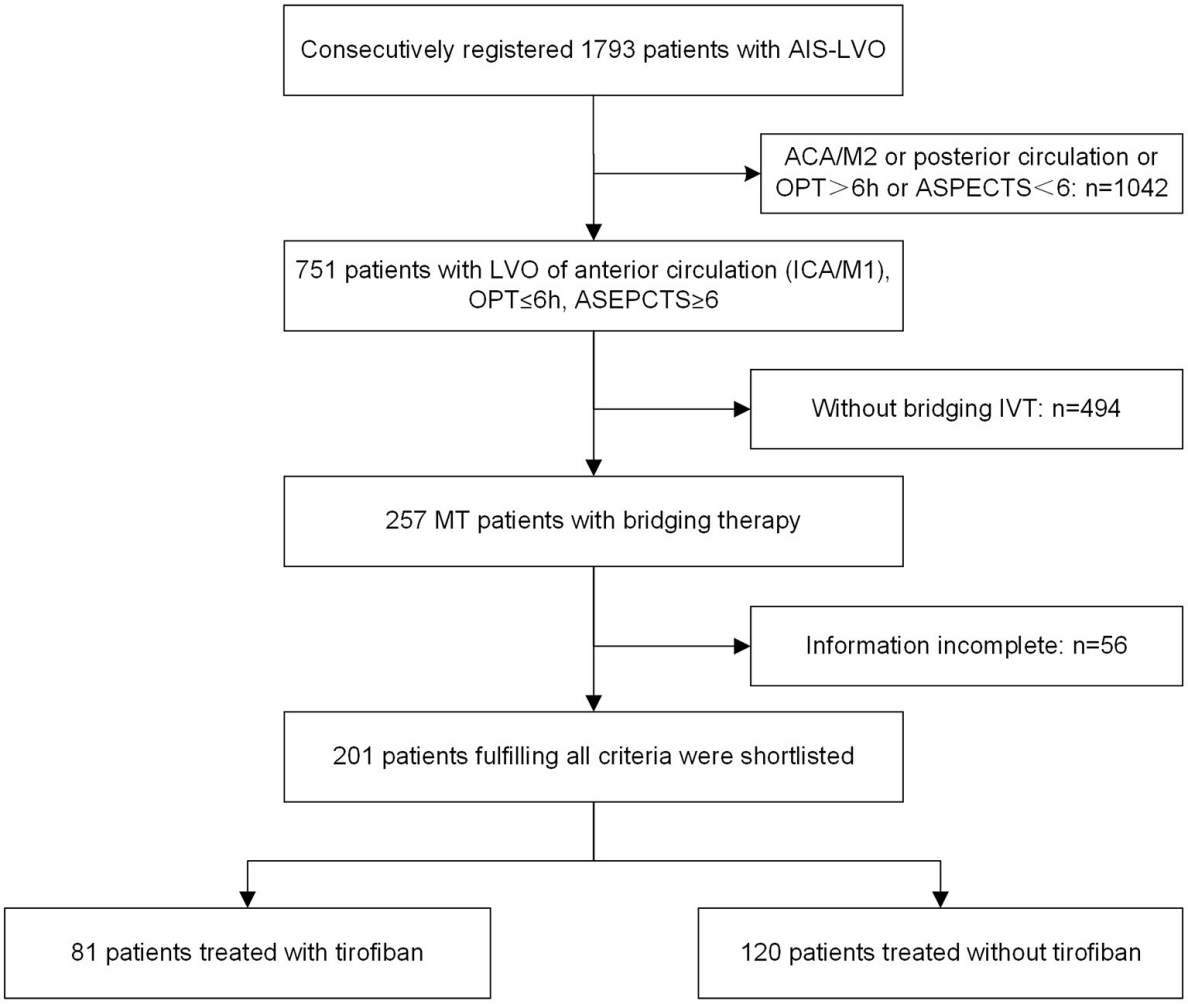
Flowchart. ACA, anterior cerebral artery; AIS, acute ischemic stroke; ASPECTS, Alberta Stroke Program Early CT Score; ICA, internal carotid artery; IVT, intravenous thrombolysis; LVO, large vessel occlusion; M1, M1 segment; M2, M2 segment; MT, mechanical thrombectomy; and OPT, onset-to-puncture time.

A total of 1,793 consecutive patients who underwent endovascular treatment were initially recruited from the ANGEL-ACT registry, of which 201 were subsequently shortlisted based on the above-described criteria (Figure 1); 81 in the tirofiban group and 120 in the non-tirofiban group. The median age of the 201 patients was 64 years (range 55–70 years), and 130 (64.7%) were male. Patients in the tirofiban group exhibited a significantly heavier atherosclerotic burden with respect to vascular risk factors such as hypertension (55.6% vs. 40.0%, *p* = 0.032), and were more likely to have a large-artery atherosclerotic stroke (54.3% vs. 34.2%) (Table 1). Sixty-eight patients from each group were included in the PSM analysis. The comparison of baseline characteristics between the two groups after PSM is shown in Table 2. Both groups were comparable with respect to baseline characteristics. Initial NIHSS score, IV thrombolysis, medical history, and mechanism of stroke were similar in both groups.

**Table 1:** Baseline characteristics of patients before PSM.

**Variable**	**All patients (*n* = 201)**	**Tirofiban (*n* = 81)**	**Non-tirofiban (*n* = 120)**	***P*-value**
Age, median (IQR)	64 (55–70)	62 (53–69)	65 (57–70)	0.104
Male sex, *n* (%)	130 (64.7)	52 (64.2)	78 (65.0)	1.000
Initial NIHSS score, median (IQR)	14 (11–18)	15 (13–19)	14 (11–18)	0.416
**Medical history**				
Atrial fibrillation, *n* (%)	70 (34.8)	22 (27.2)	48 (40.0)	0.071
Hypertension, *n* (%)	93 (46.3)	45 (55.6)	48 (40.0)	0.032
Diabetes mellitus, *n* (%)	29 (14.4)	16 (19.8)	13 (10.8)	0.101
Hypercholesterolemia, *n* (%)	16 (8.0)	8 (9.9)	8 (6.7)	0.435
Ischemic stroke, *n* (%)	28 (13.9)	9 (11.1)	19 (15.8)	0.409
Smoking, *n* (%)	77 (38.3)	32 (39.5)	45 (37.5)	0.883
Prior antiplatelet use, *n* (%)	23 (11.4)	9 (11.1)	14 (11.7)	1.000
Prior anticoagulant use, *n* (%)	1 (0.5)	0 (0.0)	1 (0.8)	1.000
Pre-stroke mRS score, *n* (%)				0.086
0	189 (94.0)	79 (97.5)	110 (91.7)	
1	12 (6.0)	2 (2.5)	10 (8.3)	
Stroke causative mechanism, *n* (%)				0.011
Large artery atherosclerosis	85 (42.3)	44 (54.3)	41 (34.2)	
Cardioembolism	87 (43.3)	28 (34.6)	59 (49.2)	
Other	29 (14.4)	9 (11.1)	20 (16.6)	
ASPECTS, median (IQR)	10 (8–10)	10 (8–10)	10 (8–10)	0.611
**Treatment profiles**				
General anesthesia, *n* (%)	59 (29.4)	24 (29.6)	35 (29.2)	1.000
Number of pass, median (IQR)	2.6 ± 1.7	2.7 ± 1.5	2.5 ± 1.8	0.110
Heparin during MT, *n* (%)	85 (42.3)	28 (34.6)	57 (47.5)	0.081
IA thrombolysis, *n* (%)	7 (3.5)	3 (3.7)	4 (3.3)	1.000
Permanent stenting, *n* (%)	34 (16.9)	19 (23.5)	15 (12.5)	0.055
Transfer from primary stroke center, *n* (%)	68 (33.8)	44 (36.7)	24 (29.6)	0.362
OPT time, median (IQR), min	245 (200–294)	255 (218–302)	241 (194–289)	0.056
PRT time, median (IQR), min	80 (52–125)	78 (52–128)	80 (52–119)	0.908

*ASPECTS, Alberta Stroke Program Early CT Score; IA, intraarterial; IQR, interquartile range; mRS, modified Rankin Scale; MT, mechanical thrombectomy; NIHSS, National Institutes of Health Stroke Scale; OPT, onset-to-puncture time; and PRT, puncture-to-recanalization time*.

**Table 2:** Baseline characteristics of patients after PSM.

**Variable**	**Tirofiban (*n* = 68)**	**Non-tirofiban (*n* = 68)**	***P*-value**
Age, median (IQR)	62 (54–70)	62 (53–69)	0.984
Male sex, *n* (%)	43 (63.2)	44 (64.7)	1.000
Initial NIHSS score, median (IQR)	15 (12–19)	15 (10–19)	0.877
**Medical history**			
Atrial fibrillation, *n* (%)	22 (32.4)	26 (38.2)	0.591
Hypertension, *n* (%)	36 (52.9)	25 (36.8)	0.084
Diabetes mellitus, *n* (%)	13 (19.1)	9 (13.2)	0.486
Hypercholesterolemia, *n* (%)	5 (7.4)	5 (7.4)	1.000
Ischemic stroke, *n* (%)	9 (13.2)	12 (17.7)	0.636
Smoking, *n* (%)	26 (38.2)	28 (41.2)	0.861
Prior antiplatelet use, *n* (%)	7 (10.3)	8 (11.8)	1.000
Prior anticoagulant use, *n* (%)	0 (0.0)	1 (1.5)	1.000
Pre-stroke mRS score, *n* (%)			1.000
0	66 (97.1)	65 (95.6)	
1	2 (2.9)	3 (4.4)	
Stroke causative mechanism, *n* (%)			0.608
Large artery atherosclerosis	32 (47.1)	30 (44.1)	
Cardioembolism	27 (39.7)	32 (47.1)	
Other	9 (13.2)	6 (8.8)	
ASPECTS, median (IQR)	10 (8–10)	10 (8–10)	0.802
**Treatment profiles**			
General anesthesia, *n* (%)	19 (27.9)	22 (32.3)	0.709
Number of pass, median (IQR)	3 (2–4)	2 (1–3)	0.169
Heparin during MT, *n* (%)	24 (35.3)	27 (39.7)	0.723
IA thrombolysis, *n* (%)	3 (4.4)	2 (2.9)	1.000
Permanent stenting, *n* (%)	14 (20.6)	9 (13.2)	0.361
Transfer from primary stroke center, *n* (%)	21 (30.9)	26 (38.2)	0.471
OPT time, median (IQR), min	253 (208–301)	255 (215–293)	0.969
PRT time, median (IQR), min	80 (53–130)	81 (52–117)	0.686

*ASPECTS, Alberta Stroke Program Early CT Score; IA, intraarterial; IQR, interquartile range; mRS, modified Rankin Scale; MT, mechanical thrombectomy; NIHSS, National Institutes of Health Stroke Scale; OPT, onset-to-puncture time; and PRT, puncture-to-recanalization time*.

### Safety Outcomes

Fifteen (7.5%) patients suffered sICH within 24 h after MT, and 52 (25.9%) experienced ICH. There were no significant between-group differences in the incidences of sICH, any ICH, or mortality within 3-months in the entire cohort (all *p* > 0.05). In the PSM cohort the findings were similar. Three (4.4%) patients in the tirofiban group and seven (10.3%) in the non-tirofiban group suffered sICH (*p* > 0.05). Fifteen (22.1%) patients in the tirofiban group and 25 (36.8%) in the non-tirofiban group experienced any ICH (*p* > 0.05). A total of 15 (11.0%) patients died after 3-months, 5 (7.4%) in the tirofiban group and 10 (14.7%) in the non-tirofiban group (*p* > 0.05) (Tables 3, 4).

**Table 3:** Safety and efficacy endpoints of MT patients with preceding intravenous thrombolysis before PSM.

	**All patients**	**Tirofiban**	**Non-tirofiban**	***P*-value**	**OR**	Adjusted *P*-value*	Adjusted OR*
sICH	15 (7.5)	5 (6.2)	10 (8.3)	0.785	0.72 (0.24, 2.20)	0.682	0.77 (0.21, 2.75)
Any ICH	18 (22.2)	34 (28.3)	52 (25.9)	0.412	0.72 (0.38, 1.40)	0.526	0.78 (0.36, 1.68)
Hemorrhage type, *n* (%)				0.732	NA	NA	NA
HI	33 (63.5)	13 (72.2)	20 (58.8)				
PH1	8 (15.4)	1 (5.6)	7 (20.6)				
PH2	9 (17.3)	2 (11.1)	7 (20.6)				
rPH	1 (1.9)	1 (5.6)	0 (0.0)				
IVH	0 (0.0)	0 (0.0)	0 (0.0)				
SAH	1 (1.9)	1 (5.6)	0 (0.0)				
Successful recanalization	185 (92.0)	74 (91.4)	111 (92.5)	0.769	0.86 (0.31, 2.40)	0.652	0.76 (0.23, 2.50)
Dramatic clinical improvement	65 (32.3)	27 (33.3)	38 (31.7)	0.878	1.08 (0.59, 1.97)	0.344	1.43 (0.68, 2.98)
3-month mRS, median (IQR)	3 (0–4)	3 (0–4)	3 (0–4)	0.595	1.15 (0.70, 1.89)	0.474	1.23 (0.70, 2.15)
3-month mRS 0–2	99 (49.3)	40 (49.4)	59 (49.2)	1.000	1.01 (0.57, 1.77)	0.921	1.03 (0.54, 1.97)
3-month mortality	18 (9.0)	6 (7.4)	12 (10.0)	0.620	0.72 (0.26, 2.00)	0.603	0.73 (0.23, 2.36)

*ASPECTS, Alberta Stroke Program Early CT Score; HI, hemorrhagic infarction; ICH, intracranial hemorrhage; IQR, interquartile range; IVH, intraventricular hemorrhage; mRS, modified Rankin Scale; MT, mechanical thrombectomy; NA, not applicable; NIHSS, National Institutes of Health Stroke Scale; OPT, onset-to-puncture time; OR, odds ratio; PH, parenchymal hemorrhage; rPH, remote from infarcted brain tissue; SAH, subarachnoid hemorrhage; and sICH, symptomatic intracranial hemorrhage*.

**Table 4:** Safety and efficacy endpoints of MT patients with preceding intravenous thrombolysis after PSM.

	**Tirofiban**	**Non-tirofiban**	***P*-value**	**OR**	Adjusted *P*-value^*^	Adjusted OR^*^
sICH	3 (4.4)	7 (10.3)	0.325	0.40 (0.10–1.63)	0.362	0.50 (0.11–2.25)
Any ICH	15 (22.1)	25 (36.8)	0.090	0.49 (0.23–1.04)	0.100	0.47 (0.19–1.16)
Hemorrhage type, *n* (%)			0.679	NA	NA	NA
HI	12 (80.0)	16 (64.0)				
PH1	0 (0.0)	5 (20.0)				
PH2	1 (6.7)	4 (16.0)				
rPH	1 (6.7)	0 (0.0)				
IVH	0 (0.0)	0 (0.0)				
SAH	1 (6.7)	0 (0.0)				
Successful recanalization	61 (89.7)	63 (92.7)	0.547	0.69 (0.21–2.30)	0.993	1.01 (0.22–4.68)
Dramatic clinical improvement	22 (32.4)	18 (26.5)	0.573	1.33 (0.63–2.79)	0.552	1.30 (0.54–3.13)
3-month mRS, median (IQR)	3 (0–4)	3 (0–5)	0.264	1.41 (0.78–2.56)	0.545	1.23 (0.64–2.36)
3-month mRS 0–2	34 (50.0)	31 (45.6)	0.732	1.19 (0.61–2.34)	0.744	1.14 (0.52–2.50)
3-month mortality	5 (7.4)	10 (14.7)	0.273	0.46 (0.15–1.43)	0.862	0.88 (0.22–3.55)

*ASPECTS, Alberta Stroke Program Early CT Score; HI, hemorrhagic infarction; ICH, intracranial hemorrhage; IQR, interquartile range; IVH, intraventricular hemorrhage; mRS, modified Rankin Scale; MT, mechanical thrombectomy; NA, not applicable; NIHSS, National Institutes of Health Stroke Scale; OPT, onset-to-puncture time; OR, odds ratio; PH, parenchymal hemorrhage; rPH, remote from infarcted brain tissue; SAH, subarachnoid hemorrhage; and sICH, symptomatic intracranial hemorrhage*.

* *Adjusted for age, baseline mRS score, baseline NIHSS score, ASPECTS, atrial fibrillation, hypertension, pathogenesis of stroke, heparin during MT, permanent stenting, OPT*.

### Efficacy Outcome

**Figure 2:**
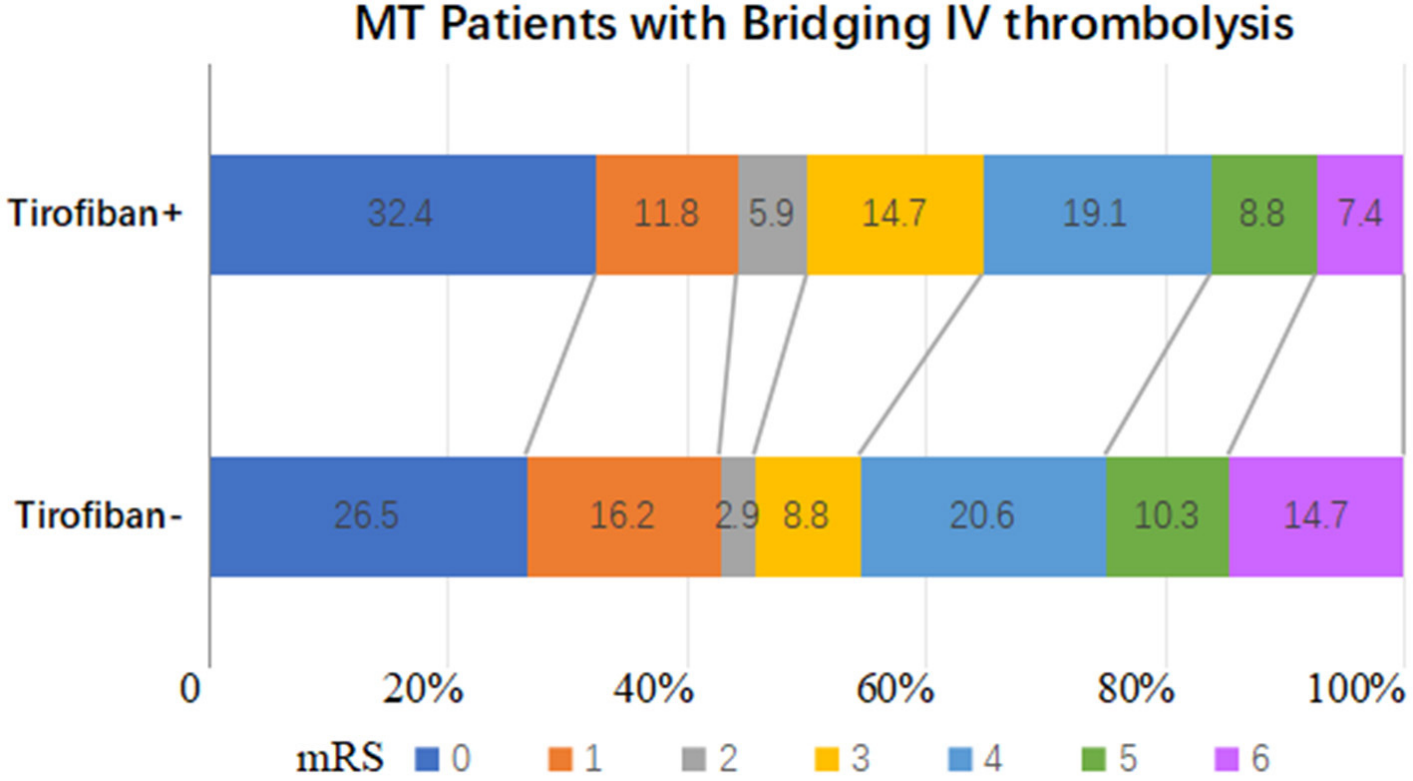
Distributions of the 3-month mRS of patients who underwent mechanical thrombectomy and preceding intravenous thrombolysis after PSM.

Overall, 185 (92.0%) patients who underwent IVT bridging therapy experienced successful recanalization, 74 (91.4%) in the tirofiban group and 111 (92.5%) in the non-tirofiban group (adjusted *p* = 0.652). The successful recanalization rates in the tirofiban group and the non-tirofiban group did not differ significantly after PSM (adjusted *p* = 0.993). In the entire cohort the median NIHSS score at 24 h post-MT was 9 (range 3–14). Sixty-five (32.3%) patients exhibited marked clinical improvement, 27 (33.3%) in the tirofiban group and 38 (31.7%) in the non-tirofiban group. At the 3-month follow-up timepoint, 99 (49.3%) patients had reached functional independence, 40 (49.4%) in the tirofiban group and 59 (49.2%) in the non-tirofiban group (Figure 2). There were no significant differences in any of the above outcomes between the two groups (all *p* > 0.05). Consistent results were observed in the PSM analysis.

## Discussion

In the current prospective registry study, low-dose tirofiban during MT with bridging IVT exhibited acceptable safety with respect to sICH and ICH. The ICH rate was lower in the tirofiban group, but not significantly before or after PSM. This suggested that low-dose tirofiban may be a safe alternative therapy during MT in patients with bridging IVT, especially those with severe *in situ* atherosclerotic stenosis, permanent stenting, or obvious endothelial damage.

Tirofiban is a non-peptide antagonist of the glycoprotein IIb/IIIa receptor, which regulates the final pathway of platelet aggregation (10). To date little high-quality research has focused on the effects of therapy with glycoprotein IIb/IIIa receptor antagonists during MT in patients with bridging IVT. Huo et al. (20) reported the safety of tirofiban in patients who underwent bridging therapy, but did not detect benefits on long-term functional outcomes. In contrast, Kellert et al. (13) concluded that tirofiban was associated with a higher risk of fatal ICH and poorer outcomes, regardless of whether preceding IVT was administered or not. Notably however, the two studies were observational studies with uncontrolled experimental designs, limited sample sizes, and heterogeneous treatment modalities, thus caution is advised when generalizing from their results.

The use of tirofiban was at the discretion of the treating physician and local practice in the present study. Consistent with previous studies, large-artery atherosclerotic stroke pathogenesis was significantly higher in the tirofiban group (*p* = 0.011) before PSM. It may be more difficult to achieve successful recanalization in patients with underlying atherosclerotic stenosis, and re-occlusion is more common, so tirofiban with or without angioplasty as an adjuvant rescue strategy may be required. This is concordant with the higher incidence of stent placement in the tirofiban group (23.5% vs. 12.5%, *p* = 0.055).

In combination therapy with intravenous thrombolysis, Zinkstok et al. (21) suggested that early intravenous administration of aspirin shortly after rt-PA was significantly associated with a higher risk of sICH in the Antiplatelet Therapy in Combination With rt-PA Thrombolysis in Ischemic Stroke trial. Based on this, the use of antiplatelet agents is not recommended within 24 h after IVT in the AHA/ASA guidelines because of the concern of increased hemorrhagic complications (15). Notably however, different inhibition modalities and biologic half-lives influence responses to medication-induced bleeding. Tirofiban is a highly selective and reversible glycoprotein IIb/IIIa receptor antagonist, and has been proven to be safe within the first 24 h after IVT (22). Low-dose tirofiban has been selectively used as rescue therapy during MT in patients with endothelial damage or *in situ* atherosclerotic stenosis in our clinical practice, and has exhibited acceptable safety. The current study preliminarily confirmed the safety of low-dose tirofiban during MT with respect to sICH and ICH in patients with preceding IVT.

The results of the current study differ from those reported by Kellert et al. (13) and Wu et al. (23) with regard to the safety of rescue tirofiban during MT. This might be due to the following reasons. One pertains to the dosage of tirofiban administration during MT. We reviewed all studies on tirofiban dosage during endovascular treatment of LVO (24). Based on this, we introduced a low-dose intra-arterial bolus of tirofiban (0.25–1.00 mg) for rapid effects on angiographic changes, followed by a continuous intravenous infusion at the lower rate of 0.1 μg/kg/min for 24 h as a standard procedure. Second, according to the specific inhibitory effect on platelet aggregation and atherothrombosis of tirofiban, we prespecified the indications for tirofiban administration during MT in the protocol. Thus, tirofiban was more selectively utilized for large-artery atherosclerotic infarction rather than cardio-embolic stroke (54.3% vs. 34.6%), which might reduce the risk of bleeding. Notably, some of the clinical characteristics of the tirofiban group differed from those of the non-tirofiban group before PSM, which may have affected outcomes. Consequently, PSM was applied to reduce the influence of confounding variables.

The current study had several limitations. First and foremost, all subjects were from an observational study. PSM analysis and a multivariable logistic regression model were used in an effort to reduce selection bias, but potential confounders cannot be ruled out despite adjustment and matching. Therefore, the results of the study need to be interpreted carefully, particularly given that the rate of sICH was lower in the tirofiban group after PSM. Another potential limitation was that all subjects were from China, which has a high prevalence of intracranial atherosclerosis (25). Thus, the results of the study may not be directly generalizable to other populations.

## Conclusion

In summary, low-dose tirofiban during MT was not associated with an increased risk of sICH or ICH in patients with preceding IVT. Further dose-escalation trials are needed to confirm its safety and efficacy.

## Data Availability Statement

The raw data supporting the conclusions of this article will be made available by the authors, without undue reservation.

## Ethics Statement

The studies involving human participants were reviewed and approved by IRB of Beijing Tiantan Hospital, Capital Medical University. The patients/participants provided their written informed consent to participate in this study. Written informed consent was obtained from the individual(s) for the publication of any potentially identifiable images or data included in this article.

## Author Contributions

ZM designed, led the study, had full access to all of the data in the study, and takes responsibility for the integrity of the data and the accuracy of the data analysis. GM and SL prepared the first draft of the report. AW and YSP did statistical analyses. All authors except AW and YSP participated in patient enrolment and collection of data. All authors critically reviewed the report and approved the final version.

## Conflict of Interest

The authors declare that the research was conducted in the absence of any commercial or financial relationships that could be construed as a potential conflict of interest. The reviewer WZ declared a shared affiliation, with no collaboration with several of the authors to the handling Editor.
